# Universal prenatal screening: a initiative from Guanajuato, Mexico to improve equity in perinatal healthcare

**DOI:** 10.3389/fmed.2023.1127802

**Published:** 2023-05-18

**Authors:** Ma de la Luz Bermudez Rojas, Virginia Medina Jimenez, Javier Ivan Manzanares Cuadros, Daniel Alberto Diaz Martínez, Nicolas Padilla Raygoza, Elia Lara Lona

**Affiliations:** ^1^State Center for Timely Prenatal Screening, Maternal and Child Hospital of Leon Guanajuato, Maternal-Fetal Unit, Institute of Public Health, Leon, Guanajuato, Mexico; ^2^Institute of Public Health, Leon, Guanajuato, Mexico; ^3^Department of Research and Technological Development, Directorate of Teaching and Research Institute of Public Health, Leon, Guanajuato, Mexico

**Keywords:** pregnancy, prenatal care, prevention, prenatal diagnosis, congenital abnormalities, preeclampsia, premature birth, pregnancy complications

## Abstract

The prenatal approach from a preventive perspective is necessary to reduce perinatal complications. A perinatal care model with a holistic and horizontal approach is required. Mexico is currently considered an emerging market economy with inequality and an economic gap that impacts the accessibility and distribution of healthcare services. Guanajuato is one of the 32 states of Mexico and represents 1.6% of the country’s surface. Strategies during the prenatal approach allow prediction, diagnosis, and anticipation of the principal causes of morbidity and mortality. Combining data from maternal characteristics and history with findings of biophysical and biochemical tests at 11 to 13 weeks of gestation can define the patient-specific risk for a large spectrum of complications that include miscarriage and fetal death, preterm delivery, preeclampsia, congenital disorders, and fetal growth abnormalities. We aim to describe the care model designed and implemented in the State Center for Timely Prenatal Screening of the Maternal and Child Hospital of Leon, Guanajuato, Mexico. Previous research showed there is a lack of information for low and middle-income countries regarding how to integrate prenatal screening strategies in the absence of resources to perform cell-free fetal DNA or biochemical serum markers in countries with emergent economies. This care model is carried out through horizontal processes where the screening is provided by trained and certified general practitioners who identify the population at risk in a timely manner for specialized care, and could help guide other Mexican states, and other countries with emergent economies with limited financial, professional, and infrastructural resources to improve prenatal care with a sense of equity, equality, and social inclusion as well as the timely evaluation of specialized perinatal care of high-risk patients.

## 1. Introduction

An accessible and effective care model for perinatal health under a predictive approach based on identifying obstetric risk factors, characterization of prenatal ultrasonographic parameters, and individualized obstetric follow-up by trained and experienced health personnel is essential to lessen perinatal morbidity and mortality, especially maternal death. Maternal and child indicators are considered parameters that reflect the quality of healthcare provided by health services in a population, such as availability, geographic and economic accessibility, acceptability, and successful coverage based on a structured referral system. Some of the sustainable development goals are to reduce the global maternal mortality ratio to less than 70 per 100,000 live births and abolish preventable deaths of newborns and children under 5 years of age. All countries aim to reduce neonatal mortality to at least as low as 12 per 1,000 live births and under 5 years mortality to at least as low as 25 per 1,000 live births by 2030 (1), currently far from possible for countries with emerging market economies. Previous research showed a lack of information for low and middle-income countries regarding the integration of prenatal screening strategies in the absence of resources to perform cell-free fetal DNA or biochemical serum markers in countries with emergent economies. Such countries should be a priority to improve maternal and child health since they represent the home of 75% of the world’s population where 62% of the world is poor (2), they have a high rate of births and morbimortality by implementing cost-effective strategies with high impact and easy access and without economic barriers that allow timely identification of risk situations. Prenatal screenings evaluate the probability of having a healthy fetus and a term of pregnancy in the best conditions. A third of the Latin American population experiences multiple barriers that hinder healthcare (3); the system recommended by PAHO/WHO called SIP (acronym in Spanish for Perinatal Information System) by using indicators to evaluate perinatal health policies, programs, and services, it is a system oriented to public health and not to individual decision-making (4). The SIP is not used in Mexico; prenatal control is based on three guiding documents: The sexual and reproductive health program (5); the NOM (acronym in Spanish for Official Mexican Standard 007) (6) and Clinic Practice Guidelines (7).

The NOM establishes to perform an ultrasound on each trimester of pregnancy by trained personnel to determine maternal and fetal well-being. The first between 11–13.6 weeks to determine gestational age, fetal vitality, and the number of fetuses; the second between 18–22 weeks, and the third between 29–30 weeks which does not contemplate the integration of risk factors and biophysical parameters proposed by other models such as the one proposed by Nicolaides et al. (8–11).

The implementation of universal prenatal screening represents many challenges such as overcoming barriers of accessibility with a lack of economic resources to provide timely attention. Therefore, we aim to describe the care model designed and implemented in CETO (acronym in Spanish for the State Center for Timely Prenatal Screening), currently the chief maternal-fetal reference unit of the Institute of Public Health in the State of Guanajuato, Mexico which provides health care mainly to the population that does not have a formal job. The care model is carried out through horizontal processes where the screening is provided by trained and certified general practitioners who identify the population at risk in a timely manner for specialized care, shortens waiting times for timely surveillance through an individualized risk estimation for specific entities such as preeclampsia, prematurity, congenital disorders, and fetal growth restriction, applicable to countries with emerging market economies with limited financial, professional, and infrastructural resources.

## 2. Context

Mexico is considered an emerging market economy (12) with many differences that persist between municipalities within regions (13). The inequality and economic gaps are very evident and impact the accessibility and distribution of healthcare services. Mexico has a fragmented healthcare system; the provision of services is granted with or without social security. Half of the population does not have social security, therefore this health care model was created to provide primary health care and universal access focused especially on those pregnant women who lack a formal job and health insurance.

In Mexico, pregnant women from rural areas with lower socioeconomic levels in marginalized environments and without health insurance, are those with the most deferrals in prenatal care (14) and at an increased risk of maternal and perinatal morbidity and mortality. The calculated maternal death ratio is 30.5 deaths per 100,000 estimated births in Mexico. Our state’s maternal death ratio is 38.8 deaths per 100,000 live births (15). The leading causes of death are preeclampsia-eclampsia (18%), obstetric hemorrhage (17.4%), and abortion (7.1%), where the most affected age group is 45 to 49 years (16). On the other hand, in 2020, 22,637 fetal deaths were registered in our country, approximating a rate of 6.7 per 10,000 women of childbearing age. Of all fetal deaths, 82.9% occurred before delivery, 15.6% occurred during delivery, and the cause of 1.5% of fetal deaths is unknown (17). The principal causes of infant mortality in Guanajuato are conditions originating during the perinatal stage. Secondary causes are due to congenital malformations, deformities, and chromosomal abnormalities, with a rate of 4.9 and 3.0 per 1,000 live births, respectively.

Guanajuato is one of the 32 states that constitute the Federal Entities of Mexico, with 6,166,934 inhabitants, which represent 4.9% of the total population of Mexico (18), it has 6,166,934 inhabitants, of which 79% had healthcare coverage; 44.8% of inhabitants were covered by the Institute of Public Health from the State of Guanajuato. In 2021, of 107,409 births, 858 infant deaths under 1 year were reported (19).

According to a national retrospective study in Mexico in 2016, Guanajuato had the highest antenatal coverage (81.6%) of the 32 Mexican states. Heredia P et al. proposed that adequacy in prenatal care consists of four fundamental aspects: (a) Skilled healthcare (antenatal care provided by a nurse or a physician), (b) Timely healthcare (initial antenatal care visit during the first trimester of pregnancy), (c) Sufficient healthcare (at least four antenatal care visits during the pregnancy), and (d) Healthcare appropriate in content (an indicator summarizing the procedures and processes of care provided during antenatal care) (20). According to a national retrospective study in Mexico, antenatal care was appropriate in 71.5% (IC 95% 69.7–73.2) of the cases.

## 3. Key programmatic elements

A screening test is a medical test or procedure performed on members (subjects) of a defined population to assess the likelihood that its members have a particular disease or outcome and to reduce morbidity or mortality through early detection when treatment may be beneficial (21). CETO model’s goal is to screen the population with a holistic, horizontal approach from the first trimester of pregnancy, or as early as possible, to reduce perinatal mortality and morbidity. Its pillars are based on screening in the first, second, and third trimesters using a multiparametric approach based primarily on the Fetal Medicine Foundation. As proposed in the model of the inversion of the prenatal care pyramid proposed by Nicolaides (11), first-trimester screening allows the prediction of entities such as fetal growth restriction, preeclampsia, preterm delivery, and aneuploidy in pregnant women with a window of opportunity corresponding to 11–13.6 weeks of gestational age for referral to prenatal care with a risk-based approach. Second-trimester screening, also termed structural or morphologic sonography, is performed between 18 and 22 weeks of gestational age. Its purpose is to detect congenital anomalies, as well as to predict preeclampsia and preterm delivery. Third-trimester screening allows early detection of late-onset placental diseases and late fetal pathologies for referral after 28 weeks of gestation. In addition, screening for infections for rapid diagnosis and optimal treatment could decrease the likelihood of complications such as preterm delivery, maternal sepsis, etc. This dynamic screening allows to identify of those high-risk patients who are candidates for receiving treatments such as progesterone, cerclage, or pessary to prevent prematurity; acetylsalicylic acid to prevent preeclampsia and/or fetal growth restriction, prompt evaluation by Perinatal Genetics if necessary and/or invasive procedures and fetal surgery in cases of fetal pathology that require it.

**Figure 1:**
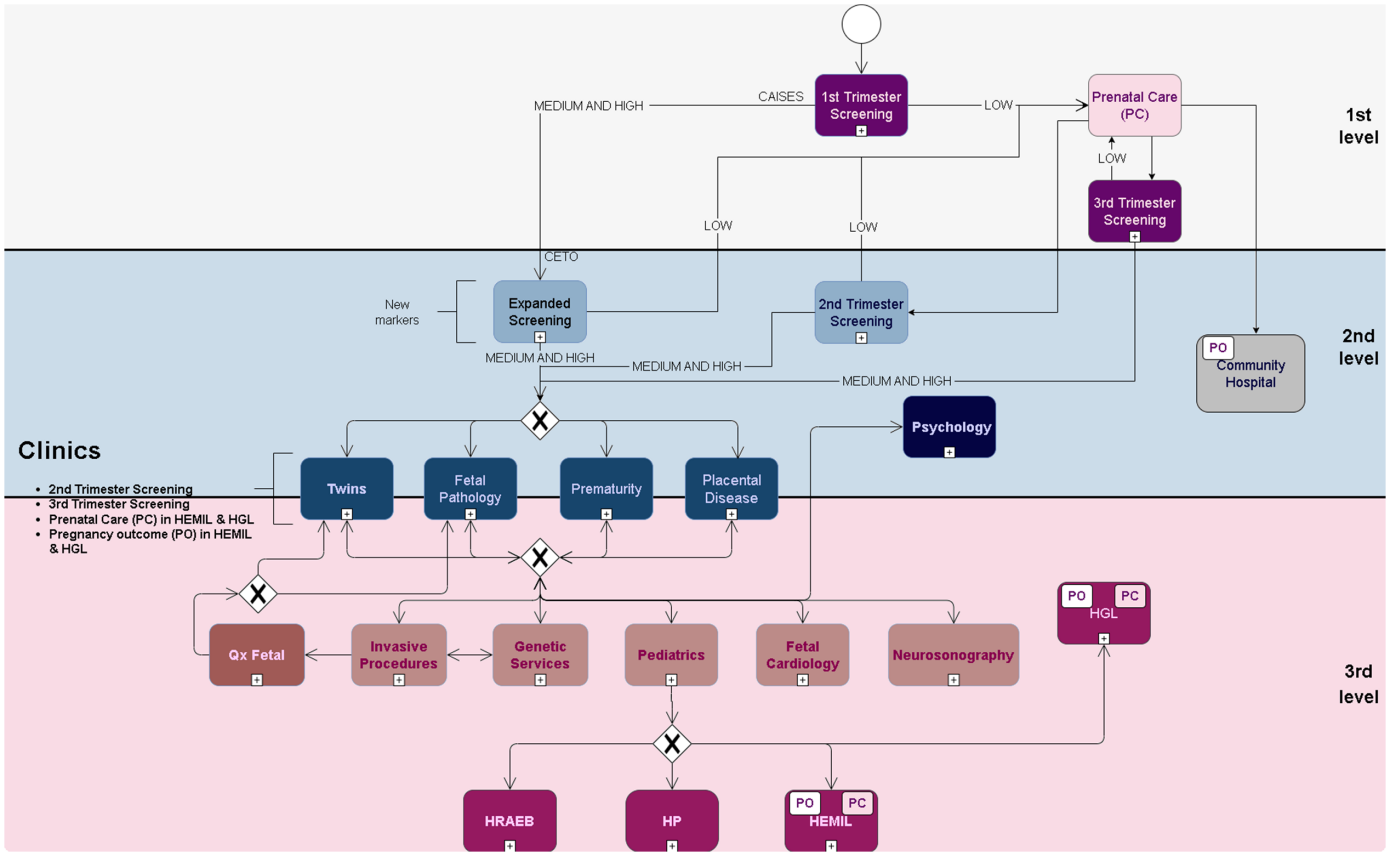
Care Flowchart and risk categorization. The model of care process of the State Center for Timely Prenatal Screening is characterized by horizontalizing perinatal care, offering screening to the pregnant population since the first trimester of pregnancy and risk evaluation for the presence of fetal aneuploidy as well as predicting risk for preeclampsia, fetal growth restriction, and preterm birth. Depending on the assigned risk, pregnant women are evaluated and followed up in the corresponding work areas where, if necessary, they are also referred to other hospital centers that belong to the National Institute of Public Health of the State of Guanajuato to continue their postnatal follow-up.

At our center, the follow-up of fetal and maternal pathologies is carried out through a specific line of work or clinics by maternal-fetal physicians. Characteristics and mode of operation are detailed below; the care flowchart and risk categorization are shown in Figure 1.

a) *Extended clinical screening* (first-trimester ultrasound markers including ductus venosus flow and tricuspid regurgitation plus detailed anatomical evaluation).
b) *Multiple gestations* (first-trimester evaluation for gestational age, chronicity, and risk categorization for the presence of aneuploidy, preeclampsia, and follow-up throughout pregnancy to identify complications, including those requiring intrauterine interventions such as a placental laser).
c) *Placental disease* (evaluation of high-risk pregnancies with the development of preeclampsia or fetal growth abnormalities, with or without prophylaxis, with acetylsalicylic acid and other abnormalities such as placenta accreta spectrum).
d) *Prematurity prevention clinic* (evaluation of high-risk pregnancies for spontaneous preterm delivery with ultrasound follow-up, including patients with progesterone, pessary, or cerclage).
e) *Soft ultrasound markers for aneuploidy* (presence of thickened nuchal fold, pyelectasis, hypoplastic nasal bone, echogenic bowel, limb shortening, choroid plexus cyst, and single umbilical artery, echogenic intracardiac focus (22). Although this marker may be questionable, follow-up of patients with soft markers is justified because performing cell-free fetal DNA is financially unviable. All cases are evaluated and followed up by specialists in maternal-fetal and perinatal genetics).
f) *Fetal pathology* (if indicated, invasive procedures or fetal surgery with perinatal follow-up).
g) *Perinatal genetic clinic* (counseling, advice, and follow-up of fetal pathology),
h) Pediatric clinic (with follow-up, coordination, and referral of newborns to different pediatric subspecialties, neurodevelopment, and nutrition).
i) Psychology (mental health assessment of pregnant patients, especially those with a diagnosis of fetal pathology to provide support and coping strategies for perinatal bereavement).
j) Second-trimester screening: Structural evaluation of patients referred from a low-risk screening in the first trimester of peripheral primary unit centers.
k) Intrauterine procedures and surgery: Such as amniocentesis, cordocentesis, chorionic villus biopsy, fetal transfusion, placental laser, etc. performed by a maternal-fetal physician with training in fetal surgery.
l) Other fetal subspecialties: Recently, due to the growing need, two specialized clinics have been created: fetal cardiology, attended by a pediatric cardiologist with training in fetal cardiology, and fetal neurology, with a maternal-fetal physician with training who, in addition to performing advanced fetal neurosonography, determines the performance of fetal MRI scans.

The objectives described in the model are evaluated annually with the following indicators: the proportion of patients screened in the first, second, and third trimesters (high and low-risk classification); the proportion of patients who dropped out of the systematic screening; the proportion of patients reported as high risk in the expanded screening; the proportion of patients that benefited from the diagnostic intervention and preventive treatment described in the care model; the proportion of patients with fetal structural alterations admitted to the model, and, the proportion of patients referred to the Genetics and Psychology clinics and proportion of fetuses with postnatal follow-up.

### 3.1. Care program development

CETO began in 2014; currently, we have performed 108,331 assessments (including the first, second, and third quarters of 2014 to June 2022). The productivity needed was calculated and derived from an estimate of the population of health jurisdiction VII with 66 medical units and an annual average of 7,540 pregnant women, and health jurisdiction VIII with 51 medical units and an annual average of 6,059 pregnant women.

The consolidation of the center went through different stages. First, the idea of the possibility of providing ultrasound and biochemical screening services for pregnant patients was supported by the state government on July 1, 2012. This model was initially called Project 78 (named after the number of jurisdictions that provided coverage in the State of Guanajuato) and was later renamed CETO. In June 2013, a visit was made by Dr. Kypros Nicolaides for counseling, with a tour of three hospitals where prenatal screening was being implemented. At the end of 2013 and in the first months of 2014, the first stage of Project 78 was implemented. This first stage served as a pilot of the initiative and included the training of a general practitioner who performed the screening in the first trimester of pregnancy at the Purisima del Rincon comprehensive care and essential health services center.

In the first half of 2014, 13 ultrasound scanners were acquired to implement and develop Project 78. Management was carried out through program Q06681, strengthening health units for surveillance and pregnancy control. In July 2014, the budget request was submitted.

From February to May, the second stage was executed. The main objective was to train general practitioners to perform first-trimester screening and triage of the population. In addition, second-trimester screenings were initiated under the supervision of an Ultrasonography specialist. All the above was carried out in the HEMIL (an acronym for Hospital Materno Infantil de León, Guanajuato) facilities.

The third stage consisted of implementing the care model proposed in the initiative. In this stage, previously trained personnel were assigned to six first-level care units (four in jurisdiction VII and two in jurisdiction VIII).

In the fourth stage, the care model was strengthened by expanding staff. Nurses, specialists, social workers, and administrative and IT personnel were hired. Two computer systems were acquired to store and generate medical reports. Also, during the fourth and fifth stages, inter-institutional collaborations were created with third-level health units (specialties) in other states, for example, the Women’s and Children’s Hospital of Querétaro, which offered surgical services.

In the fifth stage, the expansion of the Center was carried out with the management of its building and the necessary technological equipment. This building is in the HEMIL facilities. In addition, treatments for some diagnoses were incorporated into the model. The sixth stage was characterized by the expansion of the care model by adding new services.

**Figure 2:**
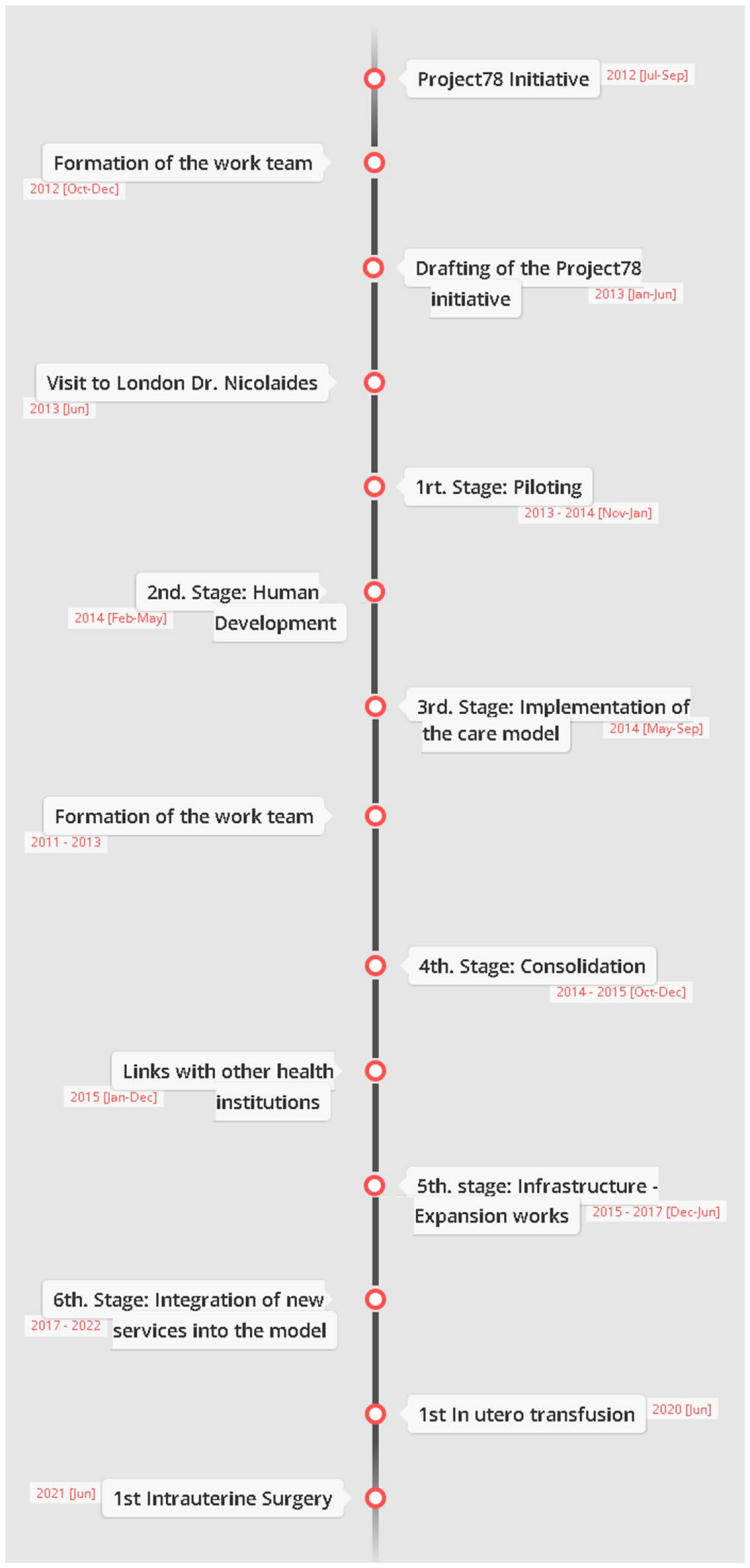
Timeline of the stages for the creation of the model of care of the State Center for Timely Prenatal Screening. This diagram shows the evolutionary process for the consolidation of the State Center for Timely Prenatal Screening which started with the Project78 initiative following through to the first fetal surgery. Our center is one of the pioneer centers in the country to perform interventions for the public sector.

The timeline of stages for the creation of the State Center for Timely Prenatal Screening care model is shown in Figure 2.

### 3.2. Operability

This multicentric center is made up of primary healthcare units with general practitioners certified by The Fetal Medicine Foundation that evaluate pregnant women from the sanitary jurisdictions VII (Casa Blanca, Las Trojes, Miguel Aleman and Floresta) and VIII (Purisima and San Francisco del Rincon) who are under continuous supervision and leadership by specialists in Obstetrics and Gynecology and Maternal-Fetal specialists from the central unit (CETO) in order to identify patients at high risk for preeclampsia, fetal growth restriction, preterm delivery, aneuploidies, birth defects, abnormal placentation, fetal infections and/or maternal comorbidities. The practitioners comprehensively protocolize the type of genetic or structural condition detected from the number of weeks of gestation, which allow an early and multidisciplinary approach in coordinated care regarding the optimal place and time of birth determined by a prompt evaluation in the Central Unit (CETO), located in the Maternal and Child Hospital of Leon, Guanajuato, Mexico. Here, there are specialists in gynecology and obstetrics, subspecialists in maternal-fetal medicine, a perinatal geneticist, a pediatrician, a fetal cardiologist, a fetal neurosonographer, a fetal surgeon, as well as psychology nurses, social workers, and administrative staff with a multidisciplinary approach.

Since biochemical markers are not available, we created a strategy with greater control to increase the detection rate during the first-trimester evaluation by incorporating the rest of the first-trimester ultrasound markers in addition to nuchal translucency (nasal bone, tricuspid flow, venous ductus flow, doppler of uterine arteries and cervical length), as well as a complementary fetal anatomical evaluation through expanded screening by a maternal-fetal subspecialist. If the patient is at low risk, she is incorporated into the low-risk patient care consultation scheme. If the patient is not at low risk, she is referred to specific care consultations; prematurity, placental pathology, soft markers of aneuploidy, fetal pathology, and perinatal genetics, except in the case of multiple pregnancy, which is evaluated by a maternal-fetal subspecialist from the onset of pregnancy to term. Simultaneously, psychologists evaluate patients in all clinics. Low-risk patients receive their prenatal check-ups in the primary care units, and their second-trimester screening at 18–22 weeks of gestation is performed in the central unit by specialized obstetrician-gynecologists. In the absence of structural anomalies and low risk for preeclampsia, and preterm delivery, patients continue their prenatal check-ups in the primary care units.

All patients categorized as high-risk pregnancies in the primary care units are evaluated in the central unit where more detailed screening is performed to assign the line of work or clinic that will provide the patient with follow-up consultations during pregnancy (multiple pregnancies, soft markers, fetal pathology including neurosonography and fetal cardiology when indicated; prevention of prematurity and placental disease). Simultaneously, a prenatal control consultation is scheduled for high-risk pregnancies to guarantee follow-up around the time of delivery at the Maternal and Child Hospital of Leon, Guanajuato (Second Level Hospital). In addition, high-risk and low-risk patients receive orientation for nutritional support, vaccinations, cervicovaginal cytology, and pregnancy education at their corresponding units.

### 3.3. Infrastructure

The center has 13 high-resolution General Electric Voluson General ultrasound units. We also have a software Viewpoint that allows us to link the ultrasound information to a clinical record to exchange medical information about the patient thereby integrating a final report of the evaluation that allows us to have a follow-up of each patient to regulate therapeutic behaviors promptly. On the other hand, we have an electronic agenda that allows us to schedule patient appointments and obtain general and individual productivity indicators for each evaluator, percentage of absences, and other healthcare quality indicators, with the added convenience of scheduling appointments by phone for the patient, or between the different interconnected units without the need of the patient to travel, making her care process more accessible and comfortable.

The interconnection between the different levels of care is achieved through the Institution’s referral. In this way, effective communication is ensured, and patients at high risk of maternal mortality or near-miss are notified to facilitate early recognition and to anticipate the optimal time and place of delivery. Fetuses with congenital anomalies are seen in a weekly face-to-face or virtual multidisciplinary session composed of pediatric subspecialists (neonatologists, cardiologists, pediatric surgeons, pediatric endocrinologists, pediatric hematologists, perinatal geneticists, among others), social workers, pediatricians, and gynecologists to identify and optimize resources around the time of delivery. Newborns and infants have follow-up visits by the pediatric hospital, the general hospital, and the high specialty hospital of Leon, Guanajuato. Newborns evaluated prenatally at our center receive follow-up, early stimulation, and neurodevelopmental evaluation at the primary care units. Newborns prenatally identified with high-risk factors for neurodevelopmental disorders such as fetal anemia, twin-to-twin transfusion, polycythemia sequence due to twin anemia, fetal growth restriction, central nervous system anomalies, congenital infections with brain tropism such as cytomegalovirus, toxoplasmosis, etc. are referred for a specialized neurodevelopmental evaluation and assessment of the possibility of neurorehabilitation and follow-up for at least the first three to 6 years of life. This is performed by the pediatrician assigned at CETO who establishes the connection between the different pediatric subspecialties and related medical areas: psychology, neurodevelopment, early stimulation, and individualized follow-up of the child’s health during infancy.

### 3.4. Casuistry

**Figure 3:**
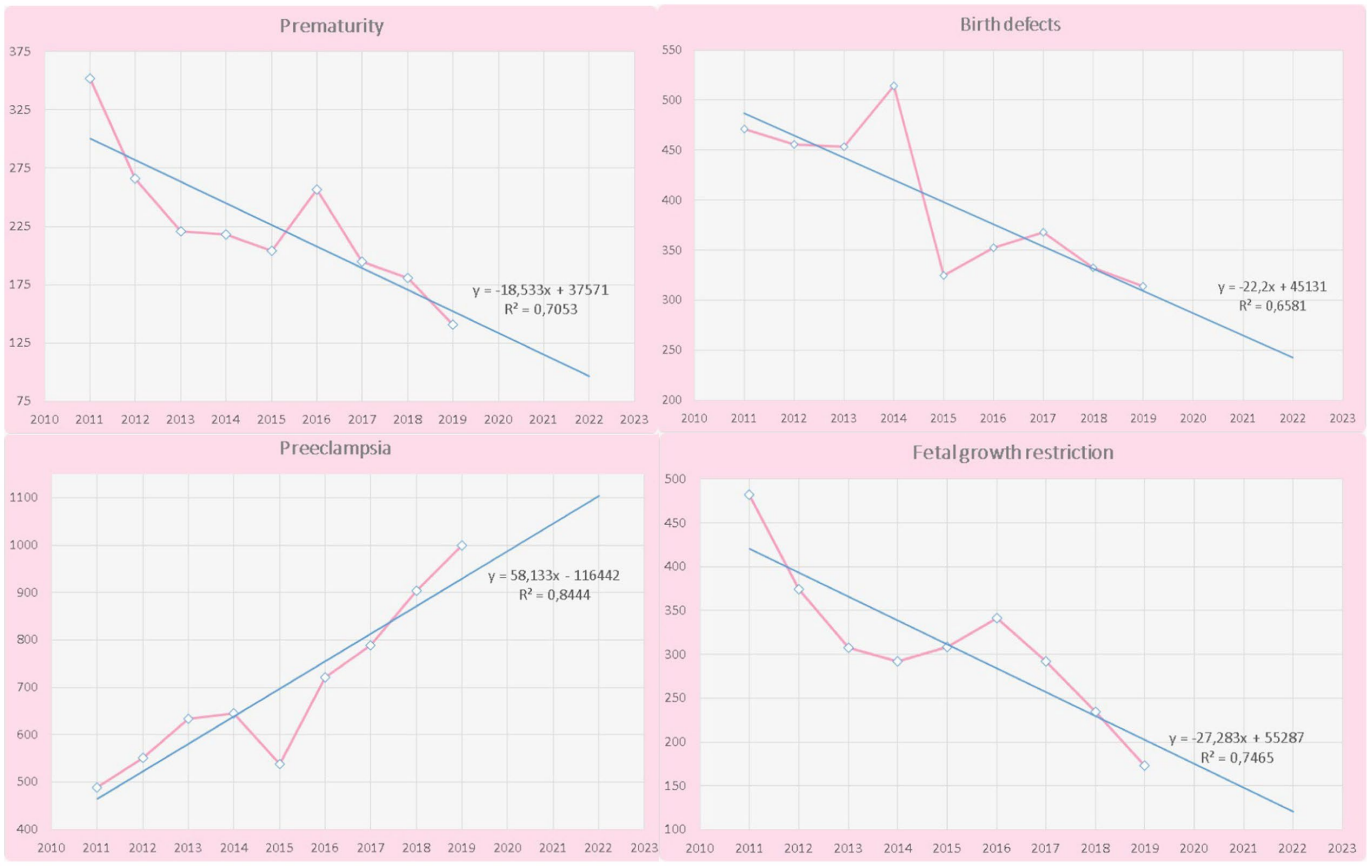
Indicators of the care model over time.

From January 2018 to December 2020, we performed 50 screenings per day, 300 screenings per week, and 1,200 screenings per month, with a total of 17,693 prenatal screenings, representing the coverage of 43.3% of the entire population (considering 40,797 pregnancies between 2018–2020 of). According to the World Health Organization, health services should be provided to 100% of the population. In our country, healthcare is divided into the public and private sectors. Our center belongs to the Public Health Institute of the State of Guanajuato, which excludes other governmental institutions. A percentage of pregnant women who do not attend complete follow-ups, either due to early loss, premature delivery, or migration to other geographic areas, is expected (20). Productivity by work areas or clinics is shown in Table 1. The coverage of the main outcomes such as preeclampsia, preterm birth, birth defects, and fetal growth restriction is shown in Table 2. The tendency of the main perinatal outcomes is shown in Figure 3.

**Table 1:** Productivity by work lines or clinics.

Work lines	2018	2019	2020	Total 2018–2020
First-trimester scan	848	688	383	1919
Second-trimester scan	1965	3,910	2,730	8,605
Third-trimester scan	886	1,049	1,148	3,083
Complementary scan (High-risk first-trimester scan)	1,142	1,109	660	2,911
Preterm prevention	1,604	2,552	1,617	5,773
Placental disease	662	655	488	1805
Multiple pregnancies	546	451	461	1,458
Soft markers	459	603	345	1,407
Fetal pathology	545	524	391	1,460
Invasive procedures (Amniocentesis, Cordocentesis, or Chorionic villus biopsy)	52	86	175	313
Pathological Uterine Arteries	836	792	621	2,249
Complementary scan (High-risk second-trimester scan)	-	24	72	96
Total of prenatal screenings	5,846	6,798	5,049	17,693

**Table 2:** Coverage of the main perinatal outcomes.

Period	Preeclampsia	Coverage %	Fetal growth restriction	Coverage %	Prematurity	Coverage %	Birth defects	Coverage %
2011	489	0.9%	482	0.9%	352	0.7%	471	0.9%
2012	551	1.0%	374	0.7%	266	0.5%	456	0.8%
2013	633	1.2%	307	0.6%	221	0.4%	453	0.8%
2014	645	1.2%	292	0.5%	218	0.4%	514	1.0%
2015	538	1.0%	308	0.6%	204	0.4%	325	0.6%
2016	720	1.3%	341	0.6%	257	0.5%	352	0.7%
2017	788	1.5%	292	0.5%	195	0.4%	368	0.7%
2018	904	1.7%	234	0.4%	181	0.3%	332	0.6%
2019	1,000	1.9%	173	0.3%	141	0.3%	314	0.6%

## 4. Discussion

Strategies carried out with a prenatal approach allow for prediction, diagnosis, and anticipation of the principal causes of mortality in pregnancy. According to a study conducted in India by Yadav A et al., female education, mass media exposure, women’s autonomy, and economic status were significantly associated with the need for maternal healthcare services (23). As reported by Yadav A et al., income and education are the most significant contributors to women’s employment of maternal healthcare services, followed by the availability of water facilities at home and in geographical regions (24). Our initiative links the most effective strategies reported in studies. From 11 to 13 weeks, the combination of maternal characteristics and historical data with the findings of biophysical and biochemical tests can define the patient’s specific risk for a broad spectrum of complications including abortion and fetal death, preterm delivery, preeclampsia, congenital disorders, and fetal growth abnormalities (11). There are a few studies published in our country regarding first-trimester screening, one of which is a study published by Oviedo-Cruz H et al. that reported an estimated detection rate of 85% and a false positive rate of 3.9%. However, the authors used biochemical and ultrasonographic markers in a Mexican population assisted by private medicine that does not resemble our population which is evaluated in the public health sector. In our case, the evaluations outnumber the tests we can perform on biochemical markers, which exemplifies the vast economic disparity that exists in our country (25). The importance of screening in the first trimester to detect congenital anomalies was demonstrated in a Mexican study by Nuñez-Sánchez G et al. The authors reported a detection rate of 83.6%, further increased to 90% by adding screening in the second trimester. The highest detection rates were obtained by screening for abdominal wall defects, nervous system alterations, cardiac defects, and skeletal anomalies (26). Early identification of patients at risk for hypertensive disorders in pregnancy, including preeclampsia and eclampsia, is advantageous to prescribe pharmacological interventions (acetylsalicylic acid) and to establish specific follow-up until obstetric outcome, a strategy validated in Mexico (8, 27–30). Lakshmy et al. defined that screening for preeclampsia by combining maternal history and biophysical or biochemical parameters, as feasible, could prevent patients from developing the complications of preeclampsia, even in low-resource settings (31).

The most frequent cause of obstetric hemorrhage is uterine atony (32). Identification of the factors that cause uterine overdistension can help to anticipate this complication. Additionally, prenatal evaluation of the placenta in high-risk patients can help to suspect the spectrum of placenta accreta which is the cause of severe postpartum hemorrhage (33). On the other hand, since 1990, most infant deaths in Mexico were due to conditions or difficulties during pregnancy, delivery, or the first month after birth, as well as congenital problems. The lethality and morbidity of congenital anomalies require early diagnosis during prenatal care (34). Screening for fetal aneuploidy at 11 to 13 weeks is possible using maternal age and fetal nuchal translucency assessed by ultrasound with a detection rate of 80% and a false positive rate of 5%, acceptable in developing countries such as Mexico, where biomarkers are not always available for financial reasons (10). Prematurity is the most unresolved problem in perinatal medicine worldwide. Premature births are responsible for 70% of neonatal deaths and 36% of infant deaths, and 25–50% of long-term neurological impairment cases in children (35). Early identification of the high-risk group for preterm spontaneous delivery before 34 weeks may be determined by an algorithm combining maternal characteristics and obstetric history with cervical length measurement to decide which patients require effective interventions such as progesterone, cerclage, or pessary (as a heroic alternative in patients refractory to progesterone and who due to gestational age are not candidates for cerclage) (11, 36). This also allows for the patient follow-up to identify the occurrence of prenatal infections that may lead to inflammation and preterm delivery to treat them promptly and may help decrease the probability of fetal brain injury (37).

To our knowledge, no previous studies have examined how to integrate all prenatal screening strategies into a single care model in Latin American countries or other middle-income countries.

The strength of our project is the horizontalization of perinatal care from an interdisciplinary approach that simultaneously includes the three levels of care, allowing other states of our country and other countries with emerging economies to replicate the broad care coverage.

Likewise, the involvement of the first level of care and specifically general practitioners guarantees greater coverage, accessibility, and cost-effectiveness of care for pregnant women; coupled with an early evaluation of the risk for a potentially developing pathology that could be prevented or reversed. This itself could be a weakness of our project, however, they are continuously trained, certified, and supervised. Additionally, gynecologist-obstetricians with master’s degrees in prenatal ultrasonography and maternal-fetal specialists evaluate the patients identified as potentially at high risk which decreases the possibility of making mistakes around perinatal care. Another weakness is that we do not yet have biochemical markers for the first trimester nor cell-free fetal DNA. However, given that our center has been equipped and strengthened over time, we do not rule out the possibility of obtaining these markers henceforth. Subsequent studies will verify the results of our care model, and this publication will serve as a precedent for the implementation and operation of this perinatal care model.

## 5. Conclusion

A fundamental part of care models is guaranteeing a care network with which the population can move effortlessly from one service to another (38). Having a horizontal model of prenatal screening makes it possible to reduce waiting times for care, as well as to provide a comprehensive approach to the mother-baby binomial, reducing delays in care, and thus reducing perinatal morbidity and mortality. CETO’s horizontal perinatal care model may serve as a guide for other Mexican states and other countries with emerging economies, with a sense of equity, equality, and social inclusion, as well as providing a timely evaluation of specialized perinatal care for high-risk patients. Other Mexican states and developing countries may replicate this care model to improve perinatal care seizing the opportunity offered by the first level of care, with regard to recruitment, accessibility, and equal conditions for pregnant women, triggering systematized screening that allows a real sense of prenatal control with a risk approach perspective.

## Data availability statement

The raw data supporting the conclusions of this article will be made available by the authors, without undue reservation.

## Author contributions

MB and VM: conceptualization. MB created and developed the model of care previously described and established in the CETO. MB and EL: methodology. JM: data analysis, design of figures, and tables. VM: writing-original draft preparation. MB, EL, DD, NP, and VM: writing-review and editing. All authors contributed to the article and approved the submitted version.

## Funding

The Institute of Public Health of the State of Guanajuato (ISAPEG) supported the fee for publishing the manuscript.

## Conflict of interest

The authors declare that the research was conducted in the absence of any commercial or financial relationships that may be construed as a potential conflict of interest.

## Publisher’s note

All claims expressed in this article are solely those of the authors and do not necessarily represent those of their affiliated organizations, or those of the publisher, the editors and the reviewers. Any product that may be evaluated in this article, or claim that may be made by its manufacturer, is not guaranteed or endorsed by the publisher.
